# First steps to understand heat tolerance of temperate maize at adult stage: identification of QTL across multiple environments with connected segregating populations

**DOI:** 10.1007/s00122-016-2674-6

**Published:** 2016-02-17

**Authors:** Felix P. Frey, Thomas Presterl, Patrick Lecoq, András Orlik, Benjamin Stich

**Affiliations:** Max Planck Institute for Plant Breeding Research, Carl-von-Linné-Weg 10, 50829 Cologne, Germany; KWS SAAT SE, Grimsehlstrasse 31, 37555 Einbeck, Germany; Group Limagrain, Am Eggenkamp 1, 48268 Greven, Germany; Group Limagrain, Fehrpart u. 80, 6710 Szeged, Hungary

## Abstract

****Key message**:**

**Dents were more heat tolerant than Flints. QTL for heat tolerance with respect to grain yield at field conditions were identified considering multiple populations and environments.**

**Abstract:**

High temperatures have the potential to cause severe damages to maize production. This study aims to elucidate the genetic mechanisms of heat tolerance under field conditions in maize and the genome regions contributing to natural variation. In our study, heat tolerance was assessed on a multi-environment level under non-controlled field conditions for a set of connected intra- and interpool Dent and Flint populations. Our findings indicate that Dent are more heat tolerant during adult stage than Flint genotypes. We identified 11 quantitative trait loci (QTL) including 2 loci for heat tolerance with respect to grain yield. Furthermore, we identified six heat-tolerance and 112 heat-responsive candidate genes colocating with the previously mentioned QTL. To investigate their contribution to the response to heat stress and heat tolerance, differential expression and sequence variation of the identified candidate genes should be subjected to further research.

**Electronic supplementary material:**

The online version of this article (doi:10.1007/s00122-016-2674-6) contains supplementary material, which is available to authorized users.

## Introduction

Maize (*Zea mays* L.) was grown on 184 million hectares in 2013 and was, thus, the second most widely cultivated crop after wheat (FAOSTAT 2014). In temperate regions of Europe, maize is of increasing importance as fodder for animal production and, lately, for biogas production (Deutsches Maiskomitee 2013).

With the progress of climate change, the global mean temperature and variance are expected to increase in the future (IPCC 2013). Lobell and Field (2007) observed a negative correlation of the yields of major crops, including maize, and an increasing global mean temperature. The effects of heat stress on plants are yield losses, growth inhibition and leaf scorching (Wahid et al. 2007), which was also reported for maize in temperate regions (Giaveno and Ferrero 2003). Especially during flowering and grain filling, heat stress has severe impacts on maize plants (Barnabás et al. 2008). Thus, breeding heat-tolerant cultivars is crucial to sustain crop production in the future (Chen et al. 2012).

Two complementary approaches are conceivable to increase heat tolerance in European maize germplasm. One possibility is to introgress exotic germplasm as described by Giaveno and Ferrero (2003). The second approach, which is described in this present study, has the potential to reduce the introgression of alleles which are associated with non-adaptedness to a temperate climate. It consists in assessing heat-tolerance variation in local germplasm and enhancing the frequency of the present positive alleles.

The molecular and physiological basis of heat tolerance in maize was studied intensively by Crafts-Brandner and Salvucci (2002), Ashraf and Hafeez (2004) and Sinsawat et al. (2004). Further, Ottaviano et al. (1991), Frova and Sari-Gorla (1994), Reimer et al. (2013) and Frey et al. (2015) investigated this question with a focus on natural variation. All these mentioned studies examined the heat tolerance of seedlings or pollen grains grown under controlled conditions. Nevertheless, experiments on seedlings can never substitute experiments on adult plants grown under field conditions (Roy et al. 2011) and can only be an auxiliary means to study the phenotypic and genotypic response to heat stress. Chen et al. (2012), Cairns et al. (2013) and Rattalino Edreira and Otegui (2013) examined heat tolerance of maize in adult stage and measured yield potential under field conditions. However, to the best of our knowledge, no previous study has used natural variation to genetically dissect heat tolerance under field conditions.

Earlier studies used different approaches to quantify the effect of a certain level of heat stress on the occurrence of phenotypic heat stress symptoms. Chen et al. (2012) and Cairns et al. (2013) described the heat tolerance of a genotype as the performance at high temperature conditions, without considering the relation of the performance at heat conditions to a control environment. Fokar et al. (1998) estimated heat tolerance in wheat by the reduction of trait values at heat conditions compared to a control condition. A more advanced approach was pursued by Mason et al. (2010) and Paliwal et al. (2012), who calculated heat susceptibility for wheat on a one-trait basis for yield components, relating the trait value of plants grown under heat conditions with their trait value at control conditions, taking into account the stress intensity at the heat conditions across all genotypes. However, to the best of our knowledge, no previous approach has been described, which includes more than two contrasting environments in the calculation of heat susceptibility.

The objectives of this study were to (I) propose a measure for heat tolerance which integrates observations from multiple levels of heat stress and assess the heat tolerance of a set of six connected segregating Dent and Flint populations for several traits and on a multi-trait level, (II) identify QTL for heat tolerance with the previously mentioned populations and (III) identify heat-tolerance candidate genes in these QTL regions.

## Material and methods

### Experimental conditions

#### Plant material and field experiments

**Fig. 1:**
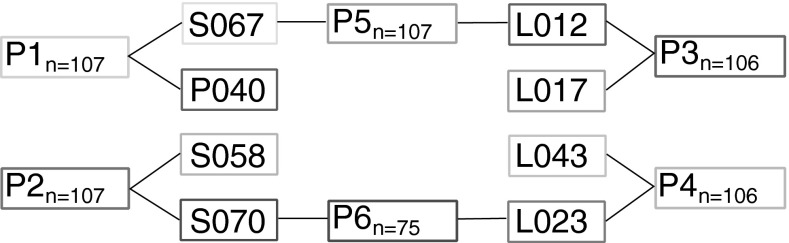
Crossing scheme used to create six segregating populations (P1–6) with number of genotypes (*N*), derived from four Dent (S067, P040, S058 and S070, in* blue*) and four Flint (L012, L017, L043 and L023, in* red*) inbred lines

This study was based on segregating populations derived from pairwise crosses of four Dent (S058, S067, S070, P040) and four Flint (L043, L017, L023, L012) maize inbred lines from the University of Hohenheim (Andersen et al. 2005). The eight inbred lines have been selected from an experiment with 74 European maize inbreds in hydroponic culture by their tolerant and susceptible phenotypic reaction upon high temperatures during seedling stage (Reimer et al. 2013) and were in detail characterized for their heat tolerance during seedling stage by Frey et al. (2015). The inbreds have been crossed pairwisely to create two Dent × Dent, two Flint × Flint and two Dent × Flint F_1_ genotypes (Fig. 1). The F_1_ genotypes were further self-pollinated resulting in six segregating populations comprising between 75 and 107 F_3:5_ genotypes and with a total of ${N=608}$ genotypes.

The genotypes were grown in field trials in summer 2012 at four locations, supervised by the plant breeding companies Limagrain (Chappes, France) and the KWS Saat AG (Einbeck, Germany), comprising two locations with standard conditions in Germany, namely Greven and Einbeck, and two locations with heat conditions, namely Zsombó (Hungary) and Monselice (Italy) (Table 1). The trials at each location were replicated twice, where each replication comprised six neighbouring subexperiments, which were outlined in alpha lattices. Each segregating population was assigned to one subexperiment. The genotypes were planted in two-row plots with 65–110 seeds per plot and a plot area between 9 and 10.5 m^2^. The eight parental inbred lines were included as standards, one time in each subexperiment. At the locations with heat conditions, plots were irrigated upon necessity by drip irrigation at Monselice and by spray irrigation at Zsombó to avoid drought stress. Further agronomic field treatments were done similarly at all locations. Air temperature and relative air humidity were recorded at 1.50 m height in all experimental fields.

**Table 1:** Experimental conditions at four field locations

Condition	Standard		Heat	
Location	Einbeck	Greven	Monselice	Zsombó
Breeding company	KWS	Limagrain	KWS	Limagrain
GPS coordinates	51$^\circ$49′N, 9$^\circ$52′E	52$^\circ$6′N, 7$^\circ$36′E	45$^\circ$13′N, 11$^\circ$45′E	46$^\circ$19′N, 19$^\circ$58′E
Meter above see level (m)	112	45	9	75
Seeds per plot	110	105	80	65
Plot area [m^2^]	9	10	9.3	10.5
Sowing date	30 April	2 May	26 April	9 May
Growing degree days	899	1136	1588	1390
Mean flowering time	3 August	2 August	3 July	14 July
Hours above 35 $^\circ$C during flowering^a^	0	0	76	34

$^{\rm a}$ 1 week before until 1 week after mean flowering

The number of days after planting when 50 % of the plants of a plot showed male (MF) and female flowering (FF), respectively, were assessed. Furthermore, data for leaf scorching (LS) of young leaves before flowering from 1 (weak damage) to 9 (strong damage) were collected. Total grain fresh yield (FY) was assessed by machine harvesting at physiological maturity, where grain moisture (GM) was measured by near infrared spectroscopy. MF, FF, GM and FY were determined by the respective plant breeding company, LS was assessed by the author of this paper. Grain dry yield per hectare (DY) at 15 % grain moisture was calculated. The anthesis silking interval (ASI) was calculated with ${\rm FF}-{\rm MF}$. Growing degree days (GDD) at each location were calculated using the model of McMaster and Wilhelm (1997):

1 $$\begin{aligned} {\rm GDD}= & {} \frac{(T_{\rm max}+T_{\rm min})}{2}-T_{\rm base}, \end{aligned}$$

where $T_{\rm max}$ and $T_{\rm min}$ were the minimum and maximum day temperature, respectively, and $T_{\rm base}$ the base temperature 10 $^\circ$C.

#### Genotyping

The parental inbred lines of the populations were genotyped with a set of 56,110 single nucleotide polymorphism (SNP) markers using a 50K SNP array (Ganal et al. 2011). Out of these SNPs, a total of 161 SNP markers were selected to genotype the individuals of the six segregating populations. For each population, between 47 and 77 markers were chosen (60 for population 1, 47 for population 2, 75 for population 3, 64 for population 4, 67 for population 5 and 77 for population 6) being polymorphic between the two parents of each population and not showing heterozygosity in either parental line. SNP marker selection was optimized for equal distribution across the physical map (due to the unavailability of a genetic map at that time) and the overlapping of markers between populations. The selected SNP markers were genotyped using KASP marker technology by TraitGenetics GmbH (Gatersleben, Germany) in the respective populations.

### Statistical analysis

#### Phenotypic data

*Adjusted entry means calculation* To estimate the environmental error effect present in each subexperiment, we used mixed model (2) with data of each trait collected for the standard genotypes, i.e. the parental inbreds, at each of the four locations separately:

2 $$\begin{aligned} Y_{ bprs}= & {} \mu + S_{ s} + R_{ r} + P_{ pr} + B_{ bpr} + e_{ bprs}, \end{aligned}$$

where $Y_{ bprs}$ was the phenotypic observation of the $s{\rm th}$ standard in the $r{\rm th}$ replication, the $p{\rm th}$ subexperiment, and the $b{\rm th}$ incomplete block, $\mu$ the general mean, $S_{s}$ the effect of the $s{\rm th}$ standard, $R_{ r}$ the effect of the $r{\rm th}$ replication, $P_{ pr}$ the effect of the $p{\rm th}$ subexperiment nested in the $r{\rm th}$ replication, $B_{ bpr}$ the effect of the $b{\rm th}$ incomplete block, nested in the $p{\rm th}$ subexperiment nested in the $r{\rm th}$ replication and $e_{ bprs}$ the residual error term. The standard factor $S_{ s}$ was not of primary interest in this analysis and was considered as a random term, just as the block effect $B_{ bpr}$. The replication effect $R_{ r}$ was set as fixed, because of the small numbers of replications per location. $P_{ pr}$ was planned to be estimated and considered as a fixed effect. The estimated subexperiment effect $\hat{P}_{ pr}$ was subtracted from the phenotypic observations of all genotypes in the corresponding subexperiment.

To calculate adjusted entry means (AEM) for each trait of the genotypes at each location, the above-mentioned adjusted phenotypic observations were analysed with model (3) at each location separately,

3 $$\begin{aligned} Y_{ bipr}= & {} \mu + G_{ i} + R_{ r} + B_{ bpr} + e_{ bipr}, \end{aligned}$$

where $Y_{ bipr}$ was the adjusted phenotypic observation for the $i{\rm th}$ genotype in the $b{\rm th}$ block of the $p{\rm th}$ subexperiment within the $r{\rm th}$ replication. $G_{i}$ denoted the fixed effect of the $i{\rm th}$ genotype and $e_{ bipr}$ the residual error term.

AEM across locations with the same condition, i.e. standard and heat, were estimated using model (4),

4 $$\begin{aligned} Y_{ bijpr}= & {} \mu + L_{j} + R_{ jr} + B_{ bjpr} + G_{i} + e_{ bijpr}, \end{aligned}$$

where $Y_{ bijpr}$ was the adjusted phenotypic observation for the $i{\rm th}$ genotype in the $b{\rm th}$ block of the $p{\rm th}$ subexperiment within the $r{\rm th}$ replication at the $j{\rm th}$ location. $L_{j}$ was the effect of the $j{\rm th}$ location within the respective condition, namely Einbeck and Greven for standard conditions, Monselice and Zsombó for heat conditions, respectively. $R_{ jr}$ was the effect of the $r{\rm th}$ replication nested in the $j{\rm th}$ location, $B_{bjpr}$ was the effect of the $b{\rm th}$ block nested in the $p{\rm th}$ subexperiment nested in $r{\rm th}$ replication nested in the $j{\rm th}$ location. $G_{i}$ was the effect of the $i{\rm th}$ genotype, which was estimated to receive AEM for the genotypes in each condition. $e_{bijpr}$ was designated as the residual error term. $G_{i}$, $L_{j}$ and $R_{jr}$ were set as fixed and the block effect $B_{bjpr}$ was regarded as random.

To calculate AEM of the traits for each location across genotypes and to assess the significance of the condition effect (standard vs. heat conditions), model (5) was used,

5 $$\begin{aligned} Y_{bcijpr}= & {} \mu + C_{c} + L_{cj} + R_{cjr} + B_{bcjpr} + G_{i} + \nonumber \\&(G.L)_{cij} + (C.G)_{ci} + e_{bcijpr}, \end{aligned}$$

where $Y_{bcijpr}$ was the adjusted phenotypic observation of the $i{\rm th}$ genotype in the $b{\rm th}$ block of the $p{\rm th}$ subexperiment within the $r{\rm th}$ replication nested in the $j{\rm th}$ location in the $c{\rm th}$ condition. $C_{c}$ was the effect of the $c{\rm th}$ condition, $L_{cj}$ was the effect of the $j{\rm th}$ location in the $c{\rm th}$ condition, $R_{cjr}$ was the effect of the $r{\rm th}$ replication nested in the $j{\rm th}$ location in the $c{\rm th}$ condition, $B_{bcjpr}$ was the effect of the $b{\rm th}$ block of the $p{\rm th}$ subexperiment within the $r{\rm th}$ replication nested in the $j{\rm th}$ location in the $c{\rm th}$ condition, $(G.L)_{cij}$ was the interaction between the $i{\rm th}$ genotype and the $j{\rm th}$ location in the $c{\rm th}$ condition and $(C.G)_{ci}$ was the interaction between the $i{\rm th}$ genotype and the $c{\rm th}$ condition. $e_{bcijpr}$ was designated as the residual error term. $C_{c}$ and $L_{cj}$ were regarded as fixed, while all other effects were regarded as random. AEM for $L_{cj}$ were estimated. Traits with a significant $C_{c}$ effect were regarded as heat-dependent traits.

*Heritability* Genotypic $\sigma ^{2}_{{\rm g}\,j}$ and error $\sigma ^{2}_{{\rm e}\,j}$ variance components for each location *j* were calculated using model (3) with a random genotype $G_{i}$ effect. For each trait, the broad sense heritability ($H^{2}_{j}$) (cf. Becker 2011; Hallauer et al. 2010) of the observations of each location *j* was calculated considering the number of replications per location (2).

Modifying the genotype model term $G_{i}$ of model (3) enabled the calculation of specific genotypic $\sigma ^{2}_{{\rm g}\,jp}$ and error $\sigma ^{2}_{{\rm e}\,jp}$ variance components for each population *p* and location *j*. Therefore, the $G_{i}$ effect of model (3), was substituted with a $(G.P)_{ip}$ interaction effect of the $i{\rm th}$ genotype and the $p{\rm th}$ population (cf. Horn et al. 2013), which was set as random. The broad sense heritability for population *p* and location *j* ($H^{2}_{jp}$) was calculated based on the population-specific $\sigma ^{2}_{{\rm g}\,jp}$ and $\sigma ^{2}_{{\rm e}\,jp}$.

To calculate genotypic $\sigma ^{2}_{{\rm g}\,cp}$, genotype–location interaction $\sigma ^{2}_{{\rm gl}\,cp}$ and error variance components $\sigma ^{2}_{{\rm e}\,cp}$ for each condition *c* and population *p*, model (4) was extended by a random genotype–location interaction effect $(G.L)_{ij}$ and the $G_{i}$ effect was regarded as random. Further, a random $(G.P)_{ip}$ and a random $(G.L.P)_{ijp}$ effect were added to the model, analogously as described previously. Broad sense heritability for each condition *c* and population *p* ($H^{2}_{cp}$) was calculated for each trait with the following model:

6 $$\begin{aligned} H^2_{cp}= \frac{\sigma ^2_{g\,cp}}{\sigma ^2_{{\rm g}\,cp} + \frac{\sigma ^2_{{\rm gl}\,cp}}{ {U}} + \frac{\sigma ^2_{{\rm e}\,cp}}{ {E*U}}}, \end{aligned}$$

where* U* was the number of locations per condition (2) and* E* the number of replications per locations (2). All mixed model analyses were performed using the software ASReml (Gilmour et al. 2006).

*Heat tolerance* A heat susceptibility index (HSI) was calculated in two steps for each heat-dependent trait (DY, FF, LS, MF and GM) times genotype combination. In the first step, the AEM of the genotypes at each location and the AEM of each location were adjusted by calculating the ratios $r_{ij}$ for genotype *i* and location *j* with

7 $$\begin{aligned} r_{ij} = \frac{\rm {AEM}_{ij}}{\rm {AEM}_{i \rm {Einbeck}}}, \end{aligned}$$

and the ratios $r_{j}$ for each location *j* across all genotypes with

8 $$\begin{aligned} r_{j} = \frac{L_{cj}}{L_{\rm {Einbeck}}} , \end{aligned}$$

where $\hbox {AEM}_{ij}$ was the AEM of genotype *i* at location *j*, calculated with model (3) and $\rm {AEM}_{i \rm {Einbeck}}$ the AEM for genotype *i* at the location Einbeck. $L_{cj}$ was the AEM for location *j* in condition *c* across all genotypes and $L_{\rm {Einbeck}}$ was the AEM for location Einbeck across all genotypes, calculated with model (5).

The second step consisted in a stability analysis (cf. Finlay and Wilkinson 1963). For each trait–genotype combination, a linear regression of $r_{ij}$ over $r_{j}$ was calculated:

9 $$\begin{aligned} r_{ij} = \rm {HSI}_{i} \times r_{j} + y_{i} + e_{ij} , \end{aligned}$$

where HSI$_i$ and $y_{i}$ were the slope and the* y*-intercept of the linear regression for genotype *i* and $e_{ij}$ the residual error term. Heat susceptibility of genotype *i* for the respective trait, was defined by the HSI$_i$. Pairwise Pearson correlation coefficients were calculated between the HSI of all heat-dependent traits across all genotypes. A secondary HSI was calculated, where the HSI for DY (HSI_DY_) was adjusted with the HSI for FF (HSI_FF_) as a cofactor using a linear regression. The residuals of the regression represented the HSI for the adjusted dry yield (HSI_DYA_).

For a multi-trait approach, the first two principal components (PC1 and PC2) of a principal component analysis (PCA) considering the previously calculated HSI of the traits DY (HSI_DY_), LS (HSI_LS_), GM (HSI_GM_), MF (HSI_MF_) and FF (HSI_FF_) for all genotypes were used as multi-trait measures for heat susceptibility.

#### Genotypic data

*Genetic map creation* SNP markers with a significant ($P<0.001$) deviation of that observed from the expected allele frequency were excluded from the analysis. To improve the mapping of markers, marker information of five segregating populations, which have been genotyped with the same set of molecular markers in a companion study (Horn et al. 2015), was included in the map creation. A consensus genetic linkage map was calculated chromosome-wise using the software CarthaGène (de Givry et al. 2005).

*QTL analysis* QTL for the assessed phenotypic data were detected using an iterative composite interval mapping approach (iQTLm) (Charcosset et al. 2001), implemented in the software MCQTL (cf. Bardol et al. 2013), making use of the above-described consensus linkage map. QTL analyses were conducted for PC1 and PC2 as well as for the HSI of the individual traits, HSI_DY_, HSI_LS_, HSI_GM_, HSI_MF_, HSI_FF_ and HSI_DYA_.

The analyses were performed across all populations (cf. the multipopulation analyses described in Bardol et al. 2013; Blanc et al. 2006). We took into account connections between populations through shared parental inbred lines using a kinship matrix specifying the parents of the six populations. We considered the additive effects of the eight parental inbred lines. Since the included biparental F_3:5_ populations showed a supposed heterozygosity of 25 %, the QTL analyses included further dominance effects between parental alleles of each biparental population. Genotypic probabilities were computed every 5 cM, taking into account information from neighbouring markers.* F* thresholds for each trait to detect QTL were determined by 1000 permutation tests, to correspond to a global type I risk of 5 % across populations and across the entire genome.* F* thresholds used to select cofactors were fixed at 90 % of the* F* threshold values for QTL detection, as suggested by the MCQTL software during the cofactor selection process. SNP markers associated with the respective trait were selected as cofactors by forward regression, where the minimal distance between two cofactors was 10 cM. At the end of the detection process, confidence intervals [logarithmic odds ratio drop regions (LOD)] were estimated on the basis of a 1.5 LOD unit fall.

To test if the dominance effects of each population on the respective QTL were significantly different from 0, significance (${\alpha =0.05}$) was calculated a posteriori from a normal distribution using a two-sided test (personal communication, Mangin, August 2014). The difference between the additive effects of pairs of parental alleles on the respective QTL was tested a posteriori using a multicomparison* t* test (Tukey) with ${\alpha =0.05}$.

*Candidate gene search* To identify candidate genes for heat tolerance in terms of the assessed traits, we mined genes, which were identified to be associated with the response and the tolerance to heat stress in a previous study (Frey et al. 2015) Therefore, we determined the genomic position on our QTL map of the previously mentioned genes by linear regression with information of the nearest two SNP markers. Candidate genes mapping in the identified QTL confidence intervals were designated in the following as heat-tolerance and heat-responsive candidate genes.

## Results

The growing degree days (GDD) from sowing until maturity were between 1400 and 1600 at locations with heat condition and between 900 and 1100 at the two locations with standard conditions (Table 1). Temperatures of the above 35 $^\circ$C were observed at the locations with heat conditions during flowering (1 week before until 1 week after mean flowering) for a period of 76 and 34 h, respectively, whereas temperatures did not reach 35 $^\circ$C during flowering at the locations with standard conditions.

We observed a significant ($P<0.001$) condition effect across populations for the traits LS, DY, FF, MF and GM (Table 3), where it was not significant for ASI. Despite the general increase of LS and decrease of DY at the location with heat compared to locations with standard conditions, we observed that Dent × Dent populations (populations 1 and 2) showed a lower increase and decrease of LS and DY, respectively, compared to Flint × Flint populations (populations 3 and 4). The decrease in DY of Dent × Flint populations (populations 5 and 6) was in between the decrease of the intra-pool (Dent × Dent and Flint × Flint) populations.

**Table 2:** Broad sense heritability of the anthesis silking interval (ASI), leaf scorching (LS), dry grain yield (DY), time to female (FF) and male flowering (MF) and grain moisture (GM) for each location* j* across the six populations (*H* ^2^_*j*_, upper left), for each location* j* and population* p* (*H* ^2^_*jp*_, bottom), and for each condition* c* and population* p* (*H* ^2^_*cp*_, upper right)

Population	Across populations				1	2	3	4	5	6	1	2	3	4	5	6								
Trait	Einbeck	Greven	Monselice	Zsombó	Standard (Einbeck and Greven)						Heat (Monselice and Zsombó)													
ASI	0.64	0.56	0.89	0.59	0.36	0.12	0.61	0.33	0.51	0.17	0.36	0.33	0.31	0.33	0.29	0.25								
LS	0.34	0.06	0.59	0.56	0.11	0.00	0.00	0.00	0.00	0.00	0.36	0.13	0.23	0.29	0.52	0.50								
DY	0.92	0.81	0.67	0.49	0.71	0.42	0.66	0.68	0.65	0.73	0.44	0.05	0.40	0.25	0.03	0.45								
FF	0.91	0.82	0.82	0.79	0.73	0.43	0.87	0.68	0.88	0.77	0.68	0.67	0.75	0.70	0.80	0.47								
MF	0.88	0.78	0.80	0.82	0.70	0.46	0.81	0.63	0.67	0.63	0.64	0.55	0.77	0.60	0.72	0.63								
GM	0.95	0.83	0.69	0.55	0.77	0.42	0.87	0.79	0.74	0.88	0.18	0.00	0.02	0.22	0.14	0.22								

Population	1	2	3	4	5	6	1	2	3	4	5	6	1	2	3	4	5	6	1	2	3	4	5	6
Trait	Einbeck						Greven						Monselice						Zsombó					
ASI	0.61	0.55	0.74	0.76	0.68	0.16	0.57	0.15	0.77	0.58	0.76	0.52	0.93	0.91	0.90	0.96	0.93	0.87	0.48	0.57	0.42	0.64	0.66	0.59
LS	0.11	0.20	0.75	0.27	0.14	0.34	$^\dagger$	$^\dagger$	$^\dagger$	$^\dagger$	$^\dagger$	$^\dagger$	0.20	0.04	0.50	0.48	0.68	0.74	0.62	0.33	0.39	0.33	0.71	0.77
DY	0.95	0.92	0.93	0.92	0.93	0.88	0.86	0.43	0.85	0.85	0.86	0.90	0.78	0.54	0.69	0.63	0.67	0.68	0.7 0	0.00	0.41	$^\dagger$	0.12	0.72
FF	0.87	0.89	0.92	0.94	0.93	0.91	0.88	0.47	0.94	0.79	0.93	0.90	0.82	0.85	0.83	0.77	0.89	0.79	0.73	0.74	0.84	0.86	0.85	0.76
MF	0.84	0.86	0.95	0.95	0.80	0.83	0.89	0.38	0.93	0.62	0.82	0.82	0.79	0.80	0.83	0.76	0.85	0.80	0.81	0.68	0.89	0.83	0.88	0.81
GM	0.95	0.95	0.98	0.93	0.94	0.95	0.85	0.38	0.91	0.90	0.88	0.94	0.83	0.80	0.64	0.57	0.54	0.91	0.36	0.00	0.88	$^\dagger$	0.71	0.65

$^\dagger$ Insufficient data collected

**Table 3:** Population-wise means of the adjusted entry means of the genotypes under heat conditions relative to the performance under standard conditions

Heterot ic group	Dent × Dent		Flint × Flint		Dent × Flint		Condition effect
Population	1	2	3	4	5	6	
ASI	116^*^^BC^	135^***^^BC^	67^***^^A^	136^***^^C^	96^ns^ ^B^	308^***^^D^	ns
LS	178^***^^A^	161^***^^A^	222^***^^B^	240^***^^B^	236^***^^B^	238^***^^B^	^***^
DY	57^***^^D^	52^***^^C^	45^***^^AB^	43^***^^A^	kg 49^***^^BC^	50^***^^BC^	^***^
FF	71^***^^B^	70^***^^A^	71^***^^B^	72^***^^C^	71^***^^B^	72^***^^C^	^***^
MF	70^***^^B^	69^***^^A^	71^***^^C^	71^***^^C^	70^***^^B^	70^***^^B^	^***^
GM	44^***^^C^	37^***^^A^	48^***^^D^	36^***^^A^	52^***^^E^	41^***^^B^	^***^

Asterisks illustrate the significance level of a pairwise* t* test, examining the difference between heat and standard conditions per population. Letters illustrate non-paired Tukey tests between the relative heat-standard differences of the six populations. In the last column, the significance of the condition (standard and heat) effect for each trait across all populations calculated with model (5) is given. For details see "Material and methods"

^*^, ^**^, ^***^ Significant at the 0.05, 0.01 and 0.001 probability level, respectively

*ns* not significant

^A^, ^B^, ^C^, ^D^ Relative differences between heat and standard conditions of populations with the same letters are not significantly (${\alpha =0.05}$) different from each other

Broad sense heritability of the four locations across populations ($H^{2}_{j}$, Table 2, upper left) was high ($0.60$–$0.79$) to very high (${>}0.80$) for the traits MF and FF and medium ($0.40$–$0.59$) to very high for ASI. $H^{2}_{j}$ was high or very high for DY and GM at Einbeck, Greven and Monselice, whereas it was medium at Zsombó. $H^{2}_{j}$ for LS was medium at locations with heat stress and low ($0.20$–$0.39$) to very low (${<}0.19$) at locations with standard conditions. The heritability across locations with the same condition, calculated for the individual populations ($H^2_{cp}$, Table 2, upper right) was lower at heat conditions compared to standard conditions for all examined traits except LS. The heritability of population 2 ($H^2_{cp}$) was lower compared to that of the other populations for the traits ASI, DY, FF, MF and GM at locations with standard conditions and for the traits LS, MF and GM at locations with heat conditions.

**Fig. 2:**
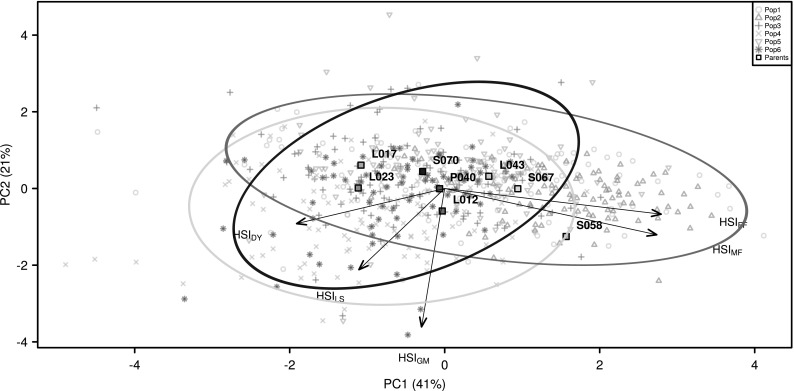
Plot of the first two principal components (PC1 and PC2) of a principal component analysis with the heat susceptibility indexes (HSI) of the time to female (FF) and male flowering (MF), leaf scorching (LS), grain moisture (GM) and dry yield (DY). The* numbers in brackets* denote the proportion of the explained variance of the respective PC of the total variance across all HSI. The* circles* represent Dent × Dent (*blue*, populations 1 and 2), Flint × Flint (*yellow*, populations 3 and 4) and Dent × Flint (*black*, populations 5 and 6) cluster

**Fig. 3:**
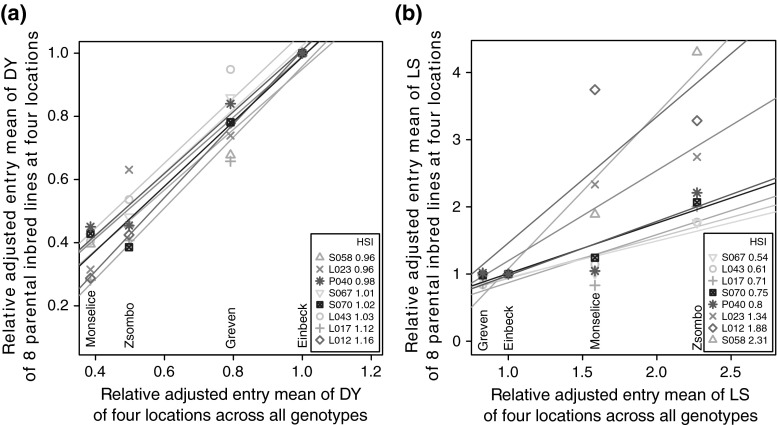
Stability analysis of the adjusted entry means (AEM) relative to that of Einbeck of **a** dry yield (DY) and **b** the leaf scorching (LS) for the parental inbred lines over the AEM of four locations across all genotypes for calculation of the heat susceptibility index (HSI)

**Fig. 4:**
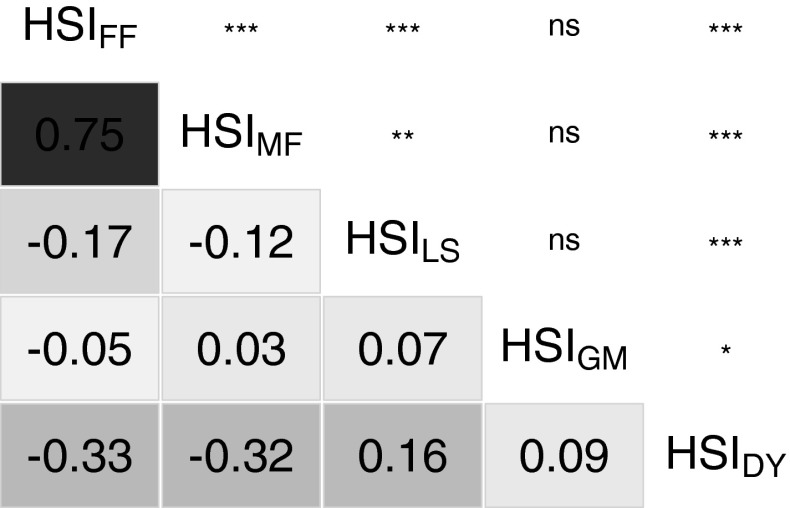
Correlations of heat susceptibility indexes (HSI) of the heat-dependent traits of female flowering (FF), male flowering (MF), leaf scorching (LS), grain moisture (GM) and dry yield (DY) with significance level (* 0.05, ** 0.01, *** 0.001, ns not significant) across all genotypes

The first two PCs of the PCA (Fig. 2) explained 41 and 21 % of the total variance of all five HSI (linear regression to calculate HSI_DY_ and HSI_LS_ of the parental inbreds, cf. Fig. 3). PC1 captured heat susceptibility with respect to yield and flowering time, with main loadings for HSI_DY_ in the negative range and for HSI_FF_ and HSI_MF_ in the positive range. PC2 had high loadings for HSI_GM_ and an intermediate high loading for HSI_LS_. In agreement with the loadings for the HSI in the PCA, we observed significant (${\alpha =0.05}$) negative correlations of HSI_DY_ with HSI_MF_ and HSI_FF_ (Fig. 4), and the correlations of HSI_DY_ with HSI_LS_ and HSI_GM_ were negligably low (${<}0.3$) although they were significant. With respect to PC1 and PC2, only overlapping clusters of Dent × Dent types (populations 1 and 2), the Flint × Flint types (populations 3 and 4) and the Dent × Flint types (populations 5 and 6) were observed (Fig. 2).

**Fig. 5:**
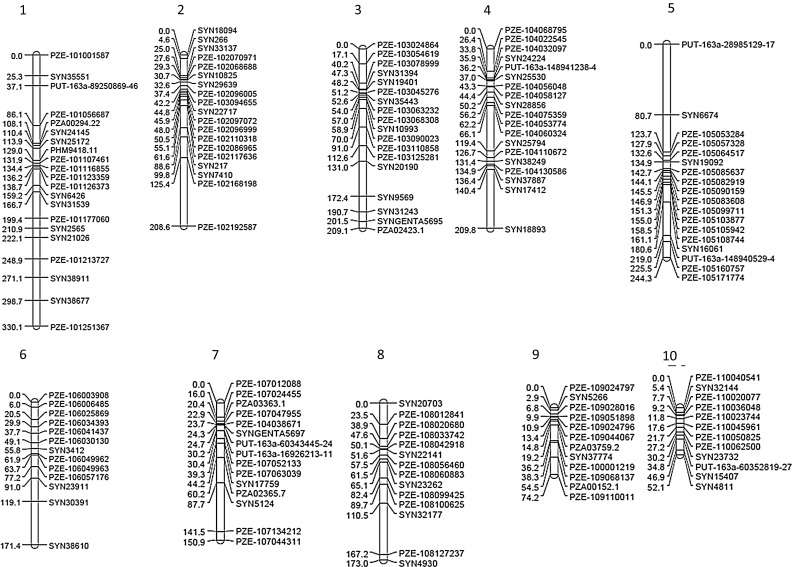
Consensus genetic linkage map with the positions of the molecular markers in cM

The consensus genetic linkage map (Fig. 5) had a total length of 1 823.5 centiMorgan (cM). The average distance was 11.3 cM and the maximum distance 83.2 cM between two markers, where markers were condensed at the centromeres of the chromosomes. Of the total of 161 markers, 21 were situated on chromosome 1, 19 on chromosome 2, 18 on chromosome 3, 19 on chromosome 4, 18 on chromosome 5, 13 on chromosome 6, 15 on chromosome 7, 14 on chromosome 8, 12 on chromosome 9 and 12 on chromosome 10.

**Table 4:** Quantitative trait loci (QTL) detected for each trait at a significance level of ${\alpha =0.05}$, with chromosome, genetic map position (cM), logarithmic odds ratio (LOD) support interval, proportion of explained variance (*R* $^2$) (%), additive effects of each parent (L043, S058, L017, L023, L012, S067, S070, P040) and dominance effects of the six (1–6) populations (1 P040xS067, 2 S070xS058, 3 L012xL017, 4 L043xL023, 5 S067xL012, 6 S070xL023)

Trait	QTL	Chr	Pos	LOD interval	*R* $^2$	Additive effect of parent								Dominance effect of population					
						L043	S058	L017	L023	L012	S067	S070	P040	1	2	3	4	5	6
HSI_DY_	Q_HSI:DYa_	2	45	33-50	12	−0.03^ABC^	−0.00^ABC^	−0.01^ABCD^	0.00^AB^	−0.03^BC^	−0.06^C^	0.03^AD^	0.10^D^	−0.15^***^	−0.01^ns^	0.00^ns^	0.04^ns^	0.02^ns^	0.06^ns^
	Q_HSI:DYb_	3	131	104-140	9	−0.01^AB^	0.05^AB^	0.04^AB^	−0.03^A^	0.07^B^	0.07^B^	−0.00^AB^	−0.18^C^	0.61^***^	0.15^ns^	−0.08^ns^	−0.37^ns^	0.15^ns^	−0.01^ns^
	Simultaneous fit				19														
HSI_DYA_	Q_HSI:DYAa_	2	45	33-55	9	−0.03^BC^	−0.01^ABC^	0.01^ABC^	0.00^ABC^	−0.03^B^	−0.05^B^	0.04^AC^	0.07^A^	−0.15^***^	−0.01^ns^	−0.02^ns^	0.03^ns^	−0.00^ns^	0.08^ns^
	Q_HSI:DYAb_	3	131	118-141	9	−0.01^AB^	0.05^AB^	0.04^AB^	−0.04^A^	0.06^B^	0.06^B^	−0.01^AB^	−0.17^C^	0.62^***^	0.17^ns^	−0.12^ns^	−0.32^ns^	0.11^ns^	−0.01^ns^
	Simultaneous fit				17														
HSI_FF_	Q_HSI:FF_	2	15	0-28	9	0.00^A^	−0.00^AB^	0.01^A^	−0.00^A^	0.00^A^	0.01^A^	0.00^A^	−0.03^B^	0.03^*^	−0.04^*^	−0.06^***^	−0.00^ns^	−0.02^ns^	0.16^ns^
HSI_LS_	Q_HSI:LS_	9	64	0-74	7	−0.04^ABC^	−0.04^ABC^	−0.22^ABC^	0.20^AB^	0.51^B^	0.29^AB^	−0.13^AC^	−0.57^C^	−0.47^ns^	−0.33^ns^	1.21^ns^	−0.09^ns^	−3.21^ns^	−0.96^ns^
HSI_MF_	Q_HSI:MFa_	2	15	0-26	9	0.01^A^	−0.01^AB^	0.00^AB^	−0.00^AB^	0.01^AB^	0.01^A^	−0.00^AB^	−0.02^B^	0.05^***^	−0.03^ns^	−0.05^***^	−0.00^ns^	−0.02^ns^	−0.01^ns^
	Q_HSI:MFb_	5	140	83-146	8	0.01^CD^	−0.00^ABCD^	−0.01^ABCD^	−0.00^AB^	0.00^ABCD^	−0.01^B^	−0.01^BC^	0.01^AD^	0.02^ns^	−0.01^ns^	0.02^ns^	0.02^ns^	0.01^ns^	0.00^ns^
	Q_HSI:MFc_	9	13	0-17	7	−0.00^AB^	−0.00^A^	−0.01^A^	0.00^AB^	−0.01^A^	0.01^B^	0.00^AB^	−0.00^AB^	0.02^ns^	0.02^ns^	−0.02^ns^	−0.00^ns^	−0.01^ns^	−0.02^ns^
	Simultaneous fit				19														
PC1	Q_PC1a_	2	10	0-22	13	0.28^AB^	−0.12^A^	0.28^AB^	0.01^A^	0.22^AB^	0.53^B^	−0.16^A^	−1.03^C^	1.59^***^	−0.55^ns^	−1.57^***^	0.08^ns^	−0.46^ns^	2.96^ns^
	Q_PC1b_	5	140	91-156	7	0.27^BC^	0.12^ABC^	−0.16^ABC^	−0.20^A^	0.05^ABC^	−0.21^A^	−0.19^AB^	0.33^C^	0.69^*^	−0.12^ns^	0.26^ns^	0.41^ns^	0.35^ns^	−0.04^ns^
	Simultaneous fit				18														

^*^, ^**^, ^***^ Significant at the 0.05, 0.01 and 0.001 probability level, respectively

^A^, ^B^, ^C^, ^D^ Additive effects of parents with same letters are not significantly different from each other

^ns^Not significant

**Fig. 6:**
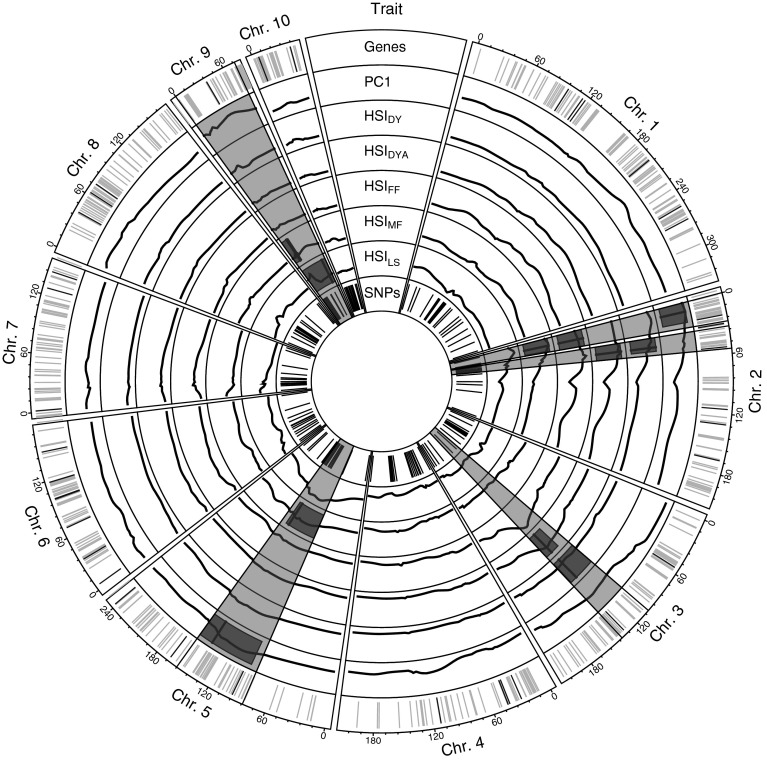
Genetic positions of heat-tolerance (*black*) and heat-responsive (*orange*) candidate genes in the quantitative trait loci (QTL) confidence intervals and flanking markers (*black*) of the QTL hot spot regions in the first track. Tracks 2–7 show logarithmic odds ratio (LOD) scores (*circumferential black*), detected QTL positions (*radial black*) and confidence intervals (*red*) of the QTL analyses for which QTL have been detected: principal component 1 (PC1) and the heat susceptibility indexes (HSI) of the traits dry yield (DY), adjusted dry yield (DYA), the time to female (FF) and male flowering (MF) and the leaf scorching (LS). QTL hot spots are denoted in* transparent red*. Genetic positions of SNP markers are shown in the* most inner circle*

We identified a total of 11 QTL (Table 4), each explaining between 7 and 13 % of the variance (*R*^2^) of the respective HSI or PC. With simultaneous fits across all QTL detected for each HSI or PC with several QTL, 19, 17, 19 and 18 % of the variance could be explained for HSI_DY_, HSI_DYA_, HSI_MF_ and PC1, respectively. The highest additive effects on QTL for HSI_DY_ and HSI_DYA_ (Q_HSI:DYa_ and Q_HSI:DYb_ as well as Q_HSI:DYAa_ and Q_HSI:DYAb_) were observed for the parental alleles of inbreds P040 and S067, which were the parental inbred lines of population 1. At the genomic position of Q_HSI:DYa_ and Q_HSI:DYAa_, the S067 allele had a negative additive effect, whereas at position of Q_HSI:DYb_ and Q_HSI:DYAb_, the P040 allele showed a negative additive effect. We observed further a highly significant dominance effect in population 1 for the previously mentioned four QTL, which was negative at Q_HSI:DYa_ and Q_HSI:DYAa_ and positive at Q_HSI:DYb_ and Q_HSI:DYAb_. A total of 6 heat-tolerance genes and 112 heat-responsive genes, identified by Frey et al. (2015), were found in the 11 QTL confidence intervals (Table 5 and Supplementary material—Table 2). Overlapping the QTL confidence intervals resulted in 5 QTL hot spots (Fig. 6), where two were located on chromosome 2 and one on chromosomes 3, 5 and 9.

**Table 5:** Heat-tolerance candidate genes within QTL confidence intervals

Gene	Chr	QTL	Description
GRMZM2G148998	2	Q_PC1a_, Q_HSI:FF_, Q_HSI:MFa_	Uncharacterized protein
GRMZM2G115658	2	Q_HSI:DYAa_	Uncharacterized protein
GRMZM2G537291	2	Q_HSI:DYAa_	Uncharacterized protein
GRMZM2G324886	3	Q_HSI:DYb_, Q_HSI:DYAb_	Calcyclin-binding protein, uncharacterized protein
GRMZM2G436710	5	Q_HSI:MFb_, Q_PC1b_	Uncharacterized protein
GRMZM2G094990	9	Q_HSI:LS_	Beta-expansin 1a, rare lipoprotein A (RlpA)-like double-psi beta-barrel

## Discussion

**Fig. 7:**
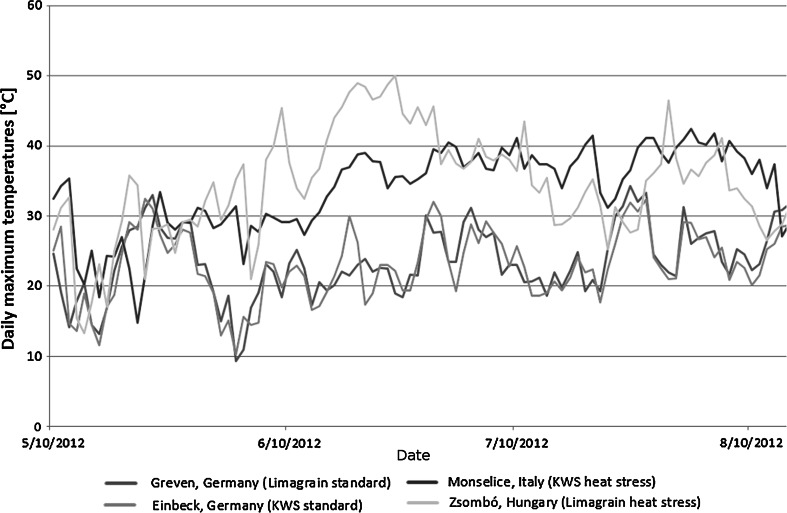
Daily maximum temperatures at four field locations during the growing period

In our experiments, the maximum daily temperatures were constantly higher at the locations with heat conditions (Monselice and Zsombó) compared to the locations with standard conditions (Einbeck and Greven), except for a heat wave in Germany in late July (Table 7). One week before until one week after the mean flowering time, temperatures exceeded 35 $^\circ$C, a total of 76 and 34 h at the locations with heat conditions, Monselice and Zsombó, respectively, whereas the temperature did not exceed 35 $^\circ$C at the locations with standard conditions, Einbeck and Greven (Table 1). Temperatures of 35 $^\circ$C during the reproductive stage of maize were stated to produce heat-related yield reduction (Hasanuzzaman et al. 2013). Maximum daily temperatures of 35 $^\circ$C and above during reproductive development of maize were associated with heat conditions (Cairns et al. 2013). In our experiments, during 15 days around flowering, we observed 0 days of maximum temperatures above 35 $^\circ$C at the locations with standard conditions and a total of 14 and 7 days of maximum temperatures above 35 $^\circ$C at the locations with heat conditions, Monselice and Zsombó, respectively. Thus, strong heat stress was present at the two locations in southern Europe in comparison to the locations in Germany and heat tolerance was successfully assessed in the year when the experiments were conducted. Besides heat stress, there might be further factors, which differed between the locations with standard conditions and the locations with heat conditions that we did not include in our analysis. We, thus, did not measure only heat tolerance but heat tolerance confounded with other factors. However, to our knowledge, the difference in temperature between the standard and the heat location were the most striking factors between them (cf. Fig. 7).

*Novel approach to assess heat susceptibility* A novel approach to calculate heat susceptibility was applied in our study to combine two characteristics of each genotype, which are involved in its response to heat stress in multiple environments. First, heat susceptibility of each genotype was defined as the difference between observations collected at heat conditions and those collected at standard conditions (Paliwal et al. 2012; Mason et al. 2010). Second, environmental stability was assessed across multiple locations (Finlay and Wilkinson 1963).

In detail, to calculate the heat susceptibility index (HSI), we first related the adjusted entry means (AEM) calculated for each genotype at each location to the AEM at the location with least heat stress, i.e. lowest temperature during the entire growing period, in this case the location Einbeck, with 899 of GDD (Table 1). With this adjustment, we removed the effect of the growth potential at optimal conditions from the observation for each genotype–location combination to account only for the relative effect of heat stress, which was the main interest of this study. The second part of the calculation of the HSI was derived from the stability analysis approach described by Finlay and Wilkinson (1963), where the stability of a genotype across environmental conditions was calculated on the basis of the performance in multiple environments. By means of these steps, we were able to combine phenotypic variation for heat tolerance in multiple environments to one index, the HSI, which can be further extrapolated to predict the performance of genotypes in other potential environments. Our approach could be appropriate to quantify the tolerance to abiotic stresses in general and could have a wide application in plant breeding experiments. However, validation with further datasets should be performed. Further, conclusions drawn from the results have to consider the data adjustment and calculations mentioned above which served to reduce the complexity of the data set.

*Heritability and assessment of traits* The heritabilities observed at the location level ($H^{2}_{j}$, Table 2, above left) were between 0.49 and 0.95 for all traits, except LS at the locations Einbeck and Greven. In another study on maize at 15 field locations with drought, heat and without stress conditions (Cairns et al. 2013), heritabilities were between 0.32 and 0.80 for grain yield, between 0.55 and 0.95 for male flowering, between 0.12 and 0.76 for the anthesis silking interval and between 0.26 and 0.90 for plant height. The moderate to very high heritabilities in our study suggested that in the current study a reliable estimation of adjusted entry means was achieved which served for calculating heat susceptibility and the detection of QTL.

We observed lower heritabilities ($H^{2}_{j}$ and $H^{2}_{cp}$) of DY at locations with heat conditions compared to heritabilities at locations with standard conditions. Cairns et al. (2013) observed the mean heritabilities of 0.84 at control, 0.64 at drought, as well as 0.50 at drought combined with heat conditions. This lower heritability at stress conditions rise from much higher error and genotype-by-location interaction variance components compared to genotypic variance components (Cairns et al. 2013). For abiotic stress studies, there is, thus, an extra need for an increased number of environments to reliably assess stress tolerance of genotypes and to study natural variation. This was achieved, in our study, with two locations in different regions of Southern Europe. The insecurity of the yield assessment of non-adapted genotypes at heat conditions strengthen furthermore the need for the application of molecular markers in the assessment of heat tolerance.

We observed heritabilities ($H^{2}_{j}$, above left, and $H^{2}_{cp}$, above right, Table 2) for LS between 0.00 and 0.34 at locations with standard conditions, whereas they were between 0.13 and 0.59 at locations with heat conditions. The lower heritability of LS at locations with standard conditions in comparison with heritabilities at locations with standard conditions for all other traits (0.12–0.95) was due to the fact that LS was rarely observed at locations without heat stress.

Besides the previously described general trends, the heritability for DY across populations ($H^{2}_{j}$, above left, Table 2) was lower at Zsombó (0.49) in comparison to the other locations (0.67–0.92). We concluded a certain insecurity of the assessment of DY at the location Zsombó. DY was calculated from the FY using GM which was assessed by near infrared spectroscopy, which demands a minimum plot yield. In Zsombó, only 65 seeds were sown per plot, in contrast to 80–110 at the other locations. FY decreased, thus, below the range, necessary for successful assessment of GM in more than 50 % of the plots, which led to missing observations. Nevertheless, the medium heritability of DY at Zsombó was sufficient to include data from this location in the analysis. Furthermore, missing data of Zsombó was compensated with data assessed at Monselice, the other location with heat conditions.

The heritability of population 2 ($H^2_{cp}$) was lower compared to that of the other populations for the traits ASI, DY, FF, MF and GM at locations with standard conditions (0.37 on average for population 2 and 0.67 on average for populations 1, 3, 4, 5 and 6) and for the traits LS, MF and GM at locations with heat conditions (0.23 on average for population 2 and 0.33 on average for populations 1, 3, 4, 5 and 6). This reduced heritability of population 2 was due to an especially low genotype variance component of this population (low variability between individuals) (supplementary material—Table 1). The low genotypic variance of population 2 was balanced using multiple populations which lead to a wide variation between individuals as a basis for the QTL analysis.

*Relation between anthesis silking interval and heat stress* In contrast to the traits LS, DY, FF, MF and GM, which were considered as heat stress dependent, we did not find a significant (*P*$<0.05$) condition effect for ASI (Table 3) and, thus, no relation of the ASI and heat stress across populations. This was in contrast to other experiments (Agrama and Moussa 1996; Bolaños and Edmeades 1996; Tuberosa et al. 2002), where the ASI was strongly increased upon drought stress. This implies that in the examined plant material in our study, a selection for flowering synchrony, i.e. reduced ASI, does not lead to increased heat tolerance.

*Influence of the reduction of the time to flowering on yield loss upon heat stress* We observed significant (*P*$<0.001$) negative correlations between HSI_DY_ and HSI_FF_ as well as HSI_MF_ (Fig. 4). Note that the correlations shown here do not state a negative correlation between the absolute values of dry yield and flowering time. Rather, genotypes which show later flowering at heat conditions compared to standard conditions also have higher yield losses at heat conditions. The analysis regarded differences between heat and standard conditions without considering absolute performance. As an avoidance mechanism, many crop plants escape heat stress by pre-maturation which is connected with preponed flowering (Hasanuzzaman et al. 2013). With our phenotypic analysis, we confirmed that preponed flowering is strongly correlated (with about 30 %) with reduced yield losses due to heat stress, which was stated previously by Hasanuzzaman et al. (2013). Thus, breeding for preponed flowering under heat conditions can help to ensure yield potential at unfavourable conditions. Hybrid testing should be performed to verify if this statement is also true in advanced breeding material. Nevertheless, earlier maturation is generally correlated with lower yields due to a shorter time to accumulate photosynthetic products. We introduced the HSI_DYA_, where the HSI_DY_ was adjusted with HSI_FF_ as a cofactor. The HSI_DYA_ can be applied to assess heat tolerance with respect to grain yield independently from the reduction of the time to flowering.

*Leaf scorching as a phenotypic marker* The correlation between HSI_DY_ and HSI_LS_ was neglegibly low (0.16) (Fig. 4). This could be explained by the low heritability of LS (Table 2) and, thus, high error of the LS assessment. Furthermore, we observed no collocation of QTL for HSI_LS_ and HSI_DY_, where a QTL for HSI_LS_ was on chromosome 9 and QTL for HSI_DY_ were on chromosomes 2 and 3. These results indicate that genetic mechanisms for LS and DY were not located at the same genomic positions. LS has, thus, limited usability as a phenotypic marker for heat tolerance in terms of yield in our populations and environments.

*Multi-trait measure for heat tolerance* The first principal component (PC) of the PC analysis represented a multi-trait measure which combined several HSI to one trait (Fig. 2). PC1 explained 41 % of the total variance and covered heat susceptibility in terms of the time to flowering and heat tolerance in terms of grain yield, as well as of leaf scorching. The QTL which were detected to be associated with PC1 (Q_PC1a_ and Q_PC1b_; Table 4) explained 18 % of the total variance of PC1 in a simultaneous fit across QTL and across populations. The highest positive influence on the PC1 was contributed by the allele of parent S067 at QTL Q_PC1a_ and by the allele of P040 at QTL Q_PC1b_. Homozygosity for allele S067 at locus Q_PC1a_ increased PC1 by a value of 0.53 and homozygosity for allele P040 at locus Q_PC1b_ increased PC1 by 0.33. Combining alleles of S067 at locus Q_PC1a_ and alleles of P040 at locus Q_PC1b_, PC1 would be increased by a total of 0.86. Furthermore, high dominance effects (significant with ${\alpha <0.001}$ at Q_PC1a_ and ${\alpha <0.05}$ at Q_PC1b_) were observed at both QTL associated with PC1 in population 1, where the parental inbreds were S067 and P040. Heterozygosity for alleles P040 and S067 at position Q_PC1a_ and Q_PC1b_ resulted in a combined dominance effect of $1.59+0.69=2.28$. Comparing homozygosity and heterozygosity at the loci associated with PC1 led to the assumption that strong heterosis for heat tolerance was present in the genetic material used in our study. As the calculation of heat tolerance in our study included phenotypic stability across conditions, the higher heat tolerance of heterozygous individuals could be attributed to higher stability across temperatures. This effect was detected previously by McWilliam and Griffing (1965), who related increased heterosis of maize hybrids at high temperatures compared to optimal growth conditions with their increased stability across growth conditions. A selection on heterozygosity with the alleles S067 and P040 at Q_PC1a_ and Q_PC1b_ would improve heat tolerance in terms of grain yield, lower leaf damages produced by heat stress and lead to an increased speed of development enabling plants to escape the strongest summer heat waves.

*Heat tolerance of Flint and Dent heterotic pools* We observed a lower yield loss and a lower increase of leaf scorching at locations with heat conditions of genotypes derived from Dent × Dent crosses in comparison with genotypes derived from Flint × Flint crosses (Table 3). Dent genotypes showed, thus, a higher heat tolerance with respect to yield and leaf scorching. Genotypes derived from interpool crosses (Dent × Flint) showed an intermediate heat tolerance with respect to the mentioned traits. To the best of our knowledge, the heat tolerance under field conditions of genotypes of the European Dent and Flint pools was not quantified previously. In a study on heat tolerance during seedling stage under controlled conditions with the inbred lines which served as parents of the populations in our study (Frey et al. 2015), no pool effect was detected. However, the low number of four Dent and four Flint inbred lines, which were phenotyped by Frey et al. (2015), did not allow a reliable conclusion on the presence of a pool effect. In the present study, heat tolerance of a total of 608 Flint, Dent and Flint × Dent genotypes from six populations was assessed. Thus, the effect on the heat tolerance of a genotype which is associated with the affiliation to a certain heterotic pool was quantified more reliably, although the genetic basis of the 608 genotypes was only eight parental inbred lines. The knowledge that genotypes derived from Dent × Dent crosses are more heat tolerant than those derived from Flint × Flint crosses is very valuable in the context of the suitability of different breeding pools for a selection on heat tolerance by plant breeders. The results were assessed with inbred lines and may be different in hybrids. However, testing inbred lines is a first step in commercial breeding programs as heritabilities are expected to be higher.

We observed significant differences between populations for heat tolerance in terms of the time to flowering (Table 3). However, those differences were not associated with the affiliation to heterotic pools. That means that, besides the reduction of the time to flowering at heat stress in general, there was no pool-specific response related to this trait. The higher heat tolerance in terms of yield of Dent genotypes compared to that of Flint genotypes might, thus, not be based on stronger reduction of the time to flowering. A possible explanation for this difference in heat tolerance is that the photosynthetically active leaf surface of Dent genotypes was less reduced by leaf scorching at heat stress compared to Flint genotypes (Table 1). As, however, the detected loci, associated with heat tolerance in terms of grain yield and in terms of leaf scorching were not overlapping (Fig. 6), the main genetic mechanisms underlying heat tolerance in terms of yield must be different. To elucidate these, we advise fine mapping of the detected QTL and functional gene studies of the candidate genes, which were located in the genome regions associated with heat tolerance (Table 5; Supplementary material—Table 2).

*Genetic linkage map* The genetic map was constructed based on molecular marker information of six segregating populations of this study and five populations of a companion study (Horn et al. 2015). This multi-population approach improved the quality of the genetic map due to a higher possibility of two markers segregating in the same population. The total length of the genetic map (1 823.5 cM) was similar to the properties of genetic maps in earlier studies in maize (e.g. Blanc et al. 2006). The average distance between molecular markers was 11.3 cM. With intervals between markers of <15 cM, any QTL is closely linked to a molecular marker, which is necessary to detect QTL and to not underestimate the magnitude of their effects (Tanksley 1993). We observed a condensation of molecular markers at the centromeres of the chromosomes on the genetic map. This was in contrast to the fact that markers were selected to be distributed evenly across the genome by physical distance. As the construction of a genetic map is always based on the probability of recombinations between loci, genetic and physical distances can vary greatly. The condensation of markers can, thus, be explained by lower recombination rates at the centromeres. This effect was described previously by Payseur and Nachman (2000). The order of the markers by their genetic positions, however, was consistent with the physical order of markers on the chromosomes.

*QTL for heat tolerance* As outlined above, heat tolerance was confounded with other envirnmental factors, but the difference in temperature between the standard and the heat locations was the most striking factor. Thus, the detected QTL represent mostly heat tolerance. Two QTL hot spots for heat tolerance with respect to grain yield (HSI_DY_ and HSI_DYA_) were identified, one on chromosome 2 and one on chromosome 3 (Fig. 6). To the best of our knowledge, QTL for heat tolerance in maize in vivo were not reported in previous studies. The latest reports on molecular markers or QTL associated with thermotolerance of maize were published in 1991 and 1994 (Ottaviano et al. 1991; Frova and Sari-Gorla 1994) and focussed on the relation of single physiological mechanisms with heat stress, i.e. the cellular membrane stability and the germination of pollen grains under heat conditions. A cluster of RFLP markers, which were associated with the injury of the pollen grain germinability and the pollen tube growth (Frova and Sari-Gorla 1994) were located at the center of chromosome 3, putatively collocating with the loci Q_HSI:DYb_ and Q_HSI:DYAb_, identified in the present study. Pollen viability is a critical mechanism involved in pollination and, consequently, seed growth. The genetic mechanisms of pollen viability, at heat stress could, thus, be a part of the reaction of maize upon heat stress with respect to grain yield. To investigate this question, the genotypes studied in this paper could be phenotyped for pollen viability traits.

Even though QTL for heat tolerance with respect to grain yield assessed on the field level were not reported previously, we observed an overlapping of the confidence intervals of the QTL detected in our study with QTL for other abiotic stresses than heat stress. The above-mentioned QTL hot spot on chromosome 2, including QTL for HSI_DY_ and HSI_DYA_, overlapped with a QTL for cold tolerance found in a meta-analysis across multiple QTL studies (Rodríguez et al. 2013) and with a QTL associated with the shoot and root dry weight and the leaf area under water stress conditions (Ruta et al. 2010). This suggests that the mentioned genomic regions might be associated with a general tolerance to abiotic stresses in maize. This, however, requires further research.

Each QTL, associated with heat tolerance with respect to different traits, detected in our study, explained between 7 and 13 % of the variance of the respective HSI or PC (Table 4). This was in accordance with the explained variances of QTL associated with abiotic stress in maize identified by Rodríguez et al. (2013) and Messmer et al. (2011). The low variance explained by single QTL in this study revealed the multigenic inheritance of heat tolerance in maize. However, with a simultaneous fit, we could explain 19 and 17 % of the total variance for HSI_DY_ and HSI_DYA_, respectively, with each of two QTL, which are located between 33 and 55 cM on chromosome 2 (Q_HSI:DYa_ and Q_HSI:DYAa_) and between 104 and 141 cM on chromosome 3 (Q_HSI:DYb_ and Q_HSI:DYAb_) (Table 4). As the statistical analyses presented in this study were based on six segregating populations, a wide genetic variation was considered. This increased the validity of the detected QTL. After validation of the genome regions in another set of environments and/or a different set of plant material, it may be profitable to invest in MAS on the previously mentioned QTL as an additional means to a traditional breeding approach.

The average absolute additive effects of the alleles of the parental inbreds P040 and S067 at the QTL for HSI_DYA_ (Q_HSI:DYAa_ and Q_HSI:DYAa_) were with 0.06 and 0.17 higher than for the other parental alleles (Table 4). to fine-map the detected QTL, i.e. to reduce their confidence intervals, we recommend performing QTL mapping including a segregating population with a higher number of progeny derived from the inbreds P040 and S067.

*Candidate genes for heat tolerance and heat response* Frey et al. (2015) identified 607 and 39 genes which were associated with the tolerance and the response upon heat stress during seedling stage under controlled conditions. To unravel the genetic mechanisms underlying heat tolerance of maize under field conditions, we examined the presence of heat-tolerance and heat-responsive genes identified by Frey et al. (2015) in seedling leaves within the QTL confidence intervals of the present study. We found that a total of 3 heat-tolerance genes and 23 heat-responsive genes were situated in the QTL regions for HSI_DY_ and HSI_DYA_ (Q_HSI:DYa_, Q_HSI:DYb_, Q_HSI:DYa_ and Q_HSI:DYb_) (Table 5; Supplementary material—Table 2). As they appear in the present study as well as in Frey et al. (2015), these genes represent genetic mechanisms which are associated with heat tolerance and heat response during both adult and seedling stage. They may, thus, be key factors for heat-related pathways in general. The heat-tolerance gene GRMZM2G324886 is of particular interest, as it was the only heat-tolerance gene, which was found in a QTL for both HSI_DY_ and HSI_DYA_ and it was already described to code for a calcicyclin-binding protein, which may be involved in calcium signalling as a response to external stress. An ortholog of this gene in rice is Os01g0757500, which was described as an HSP20-like chaperone domain containing protein and is, thus, involved in the response to heat shock. Beside its potential functional relationship with heat tolerance, our study suggests that it might be also involved in explaining phenotypic variation. This, however, needs to be studied further as follows. Due to the consideration of phenotypic variation resulting in low power to detect heat-tolerance candidate genes (Frey et al. 2015), the differential expression of GRMZM2G324886 should be verified by replicating the experiment described by Frey et al. (2015). The expression of the previously mentioned candidate gene at different heat levels could be quantified in such an experiment by quantitative real-time PCR with specific primer combination in the eight parental inbreds or even in genotypes derived from the populations used in the present study, which showed contrasting heat tolerance. After validating that GRMZM2G324886 is involved in heat tolerance, its gene sequence could be investigated with respect to polymorphisms (e.g. SNPs) between the sequences present in heat-tolerant and heat-susceptible lines, respectively, which could be the cause of differential expression. If polymorphisms are detected in the gene of interest, they would be genetically very close to the the actual QTL position detected in this study. MAS could be applied on the basis of such polymorphisms to select more heat-tolerant genotypes instead of using the flanking markers of the QTL confidence interval, which were tested in the present study and, thus, reducing the probability of recombinations between marker and QTL position in tested plants.

## Conclusion

Compared to other abiotic stresses associated with climate change (e.g. drought stress), relatively little research has been conducted on heat stress in maize. Existing studies on heat tolerance in maize focussed on a limited number of genotypes with short artificial heat stress events, rather than on the response to heat under field conditions (Cairns et al. 2013). Despite the similarities of drought and heat stress response in plants, we found that the ASI is, in contrast to drought tolerance, not related to heat tolerance. We presented a method to describe heat susceptibility without accounting for the growth potential, and which can use data of multiple environments. This approach can also be applied in studies on other abiotic stresses with multiple environments. Further, there was a lack of knowledge about the heat tolerance of either European Flint or Dent pool. This paper is a first step towards studying this point. However, a bigger set of inbred genotypes should be tested concerning their heat tolerance to verify that Dent genotypes are more heat tolerant than Flint genotypes. A further important step towards breeding of more heat-tolerant varieties is the investigation of the reaction upon heat stress of hybrid genotypes. Marker-assisted selection for heat tolerance with respect to grain yield is of great importance due to the lack of highly heritable phenotypic markers and the difficult nature of the assessment of heat tolerance (multi-environment field trials). The experiments underlying this paper can help to design experiments to further develop markers for heat tolerance in the future.

### Author contribution statement

FPF participated in the design and coordination of the study, carried out the statistical analysis and the QTL mapping and drafted the manuscript. TP, PL and AO participated in the design of and conducted the field experiments. BS conceived the study, participated in its design and coordination as well as in the statistical analysis and drafted the manuscript. All authors read and approved the final manuscript.

## Electronic supplementary material

Below is the link to the electronic supplementary material.

Supplementary material 1 (pdf 26 KB)

Supplementary material 2 (pdf 23 KB)
