# Supplementary material for: First steps to understand heat tolerance of temperate maize at adult stage: identification of QTL across multiple environments with connected segregating populations
Source: Theor Appl Genet. 2016 Feb 17;129:945–61. doi: 10.1007/s00122-016-2674-6 (PMC4835532; doi:10.1007/s00122-016-2674-6)
Supplement: Supplementary file 1 — Supplementary material 1 (pdf 26 KB) [file 122_2016_2674_MOESM1_ESM.pdf]

Table 1 Genotypic variance components of the anthesis silking interval (ASI), leaf scorching (LS), dry grain yield (DY), time to female (FF) and male flowering (MF) and grain moisture (GM) for each condition c and population p.

Population	1	2	3	4	5	6	1	2	3	4	5	6
Trait	Standard (Einbeck & Greven)						Heat (Monselice & Zsombó)					
ASI	0.89	0.21	2.93	0.69	2.64	0.36	0.34	0.44	0.26	0.29	0.31	0.19
LS	0.02	0.00	0.00	0.00	0.00	0.00	0.69	0.17	0.43	0.51	1.73	1.55
DY	1.75	0.52	1.14	1.18	0.95	1.11	0.30	0.02	0.16	0.09	0.01	0.21
FF	4.20	1.72	13.09	4.49	11.80	4.33	4.75	2.87	4.90	3.18	4.65	1.19
MF	2.89	1.33	5.67	2.36	3.45	2.47	3.51	1.16	4.53	2.13	2.73	1.63
GM	5.94	1.86	11.41	5.65	5.26	7.96	1.60	0.00	0.35	2.07	2.26	1.34

Table 2 Error variance components of the anthesis silking interval (ASI), leaf scorching (LS), dry grain yield (DY), time to female (FF) and male flowering (MF) and grain moisture (GM) for each condition c and population p.

Population	1	2	3	4	5	6	1	2	3	4	5	6
Trait	Standard (Einbeck & Greven)						Heat (Monselice & Zsombó)					
ASI	1.47	1.45	1.73	1.03	2.30	1.71	0.51	0.74	0.46	0.43	0.46	0.42
LS	0.14	0.04	0.08	0.07	0.11	0.05	1.19	1.14	1.34	1.27	1.56	1.31
DY	0.40	0.52	0.34	0.37	0.28	0.33	0.29	0.29	0.21	0.21	0.34	0.23
FF	1.33	1.66	1.68	0.95	1.42	0.78	2.22	1.31	1.58	1.39	1.05	1.03
MF	1.13	1.28	0.79	0.49	1.56	1.14	1.86	0.85	1.25	1.32	0.92	0.88
GM	1.46	2.17	1.38	1.17	1.37	0.86	3.89	2.22	13.33	6.40	11.52	1.82

Table 3 Genotype x location variance components of the anthesis silking interval (ASI), leaf scorching (LS), dry grain yield (DY), time to female (FF) and male flowering (MF) and grain moisture (GM) for each condition c and population p.

Population	1	2	3	4	5	6	1	2	3	4	5	6
Trait	Standard (Einbeck & Greven)						Heat (Monselice & Zsombó)					
ASI	0.25	0.34	0.30	0.69	0.48	0.01	0.20	0.28	0.23	0.34	0.57	0.30
LS	0.00	0.01	0.12	0.01	0.01	0.01	0.02	0.04	0.12	0.00	0.12	0.44
DY	0.66	0.43	0.48	0.36	0.45	0.15	0.19	0.10	0.06	0.09	0.21	0.05
FF	0.43	1.23	0.53	2.36	0.38	0.97	0.00	0.18	0.16	0.00	0.24	0.61
MF	0.27	0.58	1.03	1.73	0.28	0.63	0.29	0.19	0.14	0.20	0.33	0.17
GM	0.64	0.83	0.78	0.69	1.03	0.41	6.35	3.08	8.87	1.77	3.92	5.86
