# Supplementary material for: First steps to understand heat tolerance of temperate maize at adult stage: identification of QTL across multiple environments with connected segregating populations
Source: Theor Appl Genet. 2016 Feb 17;129:945–61. doi: 10.1007/s00122-016-2674-6 (PMC4835532; doi:10.1007/s00122-016-2674-6)
Supplement: Supplementary file 2 — Supplementary material 2 (pdf 23 KB) [file 122_2016_2674_MOESM2_ESM.pdf]

Table 1: Heat responsive candidate genes within QTL intervals.

Gene	Chr	QTL	Description
AC212835.3_FG008	2	Q_{PC1a}, $Q_{HSI:FF}$, $Q_{HSI:MFa}$	no information
GRMZM2G007256	2	Q_{PC1a}, $Q_{HSI:FF}$, $Q_{HSI:MFa}$	Adhesive/proline-rich protein; Uncharacterized protein
GRMZM2G016649	2	Q_{PC1a}, $Q_{HSI:FF}$, $Q_{HSI:MFa}$	Uncharacterized protein
GRMZM2G031904	2	Q_{PC1a}, $Q_{HSI:FF}$, $Q_{HSI:MFa}$	Putative uncharacterized protein
GRMZM2G037413	2	Q_{PC1a}, $Q_{HSI:FF}$, $Q_{HSI:MFa}$	Cellulose synthase catalytic subunit 11
GRMZM2G040858	2	Q_{PC1a}, $Q_{HSI:FF}$, $Q_{HSI:MFa}$	Uncharacterized protein ycf72
GRMZM2G049538	2	Q_{PC1a}, $Q_{HSI:FF}$, $Q_{HSI:MFa}$	Acyclic sesquiterpene synthase
GRMZM2G077420	2	Q_{PC1a}, $Q_{HSI:FF}$, $Q_{HSI:MFa}$	no information
GRMZM2G093526	2	Q_{PC1a}, $Q_{HSI:FF}$, $Q_{HSI:MFa}$	Uncharacterized protein
GRMZM2G125669	2	Q_{PC1a}, $Q_{HSI:FF}$, $Q_{HSI:MFa}$	Alternative oxidase
GRMZM2G153378	2	Q_{PC1a}, $Q_{HSI:FF}$, $Q_{HSI:MFa}$	Putative uncharacterized protein
GRMZM2G175447	2	Q_{PC1a}, $Q_{HSI:FF}$, $Q_{HSI:MFa}$	no information
GRMZM2G304745	2	Q_{PC1a}, $Q_{HSI:FF}$, $Q_{HSI:MFa}$	no information
GRMZM2G322819	2	Q_{PC1a}, $Q_{HSI:FF}$, $Q_{HSI:MFa}$	no information
GRMZM2G362413	2	Q_{PC1a}, $Q_{HSI:FF}$, $Q_{HSI:MFa}$	no information
GRMZM2G392125	2	Q_{PC1a}, $Q_{HSI:FF}$, $Q_{HSI:MFa}$	Uncharacterized protein
GRMZM6G859365	2	Q_{PC1a}, $Q_{HSI:FF}$, $Q_{HSI:MFa}$	Cytochrome P450 CYP87A15
GRMZM2G015727	2	$Q_{HSI:FF}$, $Q_{HSI:MFa}$	Putative uncharacterized protein
GRMZM2G051571	2	$Q_{HSI:FF}$, $Q_{HSI:MFa}$	Uncharacterized protein
GRMZM2G089596	2	$Q_{HSI:FF}$, $Q_{HSI:MFa}$	Uncharacterized protein
GRMZM2G115705	2	$Q_{HSI:FF}$, $Q_{HSI:MFa}$	Uncharacterized protein
GRMZM2G157822	2	$Q_{HSI:FF}$, $Q_{HSI:MFa}$	no information
GRMZM2G168985	2	$Q_{HSI:FF}$, $Q_{HSI:MFa}$	no information
GRMZM2G169013	2	$Q_{HSI:FF}$, $Q_{HSI:MFa}$	Uncharacterized protein
GRMZM2G177561	2	$Q_{HSI:FF}$, $Q_{HSI:MFa}$	no information
GRMZM2G060444	2	$Q_{HSI:DYa}$, $Q_{HSI:DYAa}$	Homeodomain leucine zipper family IV protein
GRMZM2G088778	2	$Q_{HSI:DYa}$, $Q_{HSI:DYAa}$	no information
GRMZM2G106092	2	$Q_{HSI:DYa}$, $Q_{HSI:DYAa}$	no information
GRMZM2G119773	2	$Q_{HSI:DYa}$, $Q_{HSI:DYAa}$	Uncharacterized protein
GRMZM2G147491	2	$Q_{HSI:DYa}$, $Q_{HSI:DYAa}$	Uncharacterized protein
GRMZM2G154437	2	$Q_{HSI:DYa}$, $Q_{HSI:DYAa}$	Uncharacterized protein
GRMZM2G154685	2	$Q_{HSI:DYa}$, $Q_{HSI:DYAa}$	no information
GRMZM2G334336	2	$Q_{HSI:DYa}$, $Q_{HSI:DYAa}$	no information
GRMZM2G520811	2	$Q_{HSI:DYa}$, $Q_{HSI:DYAa}$	no information
GRMZM5G829946	2	$Q_{HSI:DYa}$, $Q_{HSI:DYAa}$	Uncharacterized protein
GRMZM2G135990	2	$Q_{HSI:DYAa}$	Putative uncharacterized protein
GRMZM2G137964	2	$Q_{HSI:DYAa}$	Uncharacterized protein
GRMZM2G178321	2	$Q_{HSI:DYAa}$	Uncharacterized protein
GRMZM2G365815	2	$Q_{HSI:DYAa}$	Calcium-dependent protein kinase
AC209784.3_FG007	3	$Q_{HSI:DYb}$	no information
GRMZM2G007928	3	$Q_{HSI:DYb}$	Bowman-Birk type trypsin inhibitor
GRMZM2G039246	3	$Q_{HSI:DYb}$, $Q_{HSI:DYAb}$	Histidine-containing phosphotransfer protein 4; Uncharacterized protein
GRMZM2G055795	3	$Q_{HSI:DYb}$, $Q_{HSI:DYAb}$	Cellulose synthase catalytic subunit 11
GRMZM2G069511	3	$Q_{HSI:DYb}$, $Q_{HSI:DYAb}$	NHL25
GRMZM2G069694	3	$Q_{HSI:DYb}$, $Q_{HSI:DYAb}$	Uncharacterized protein
GRMZM2G133359	3	$Q_{HSI:DYb}$, $Q_{HSI:DYAb}$	no information
GRMZM2G156877	3	$Q_{HSI:DYb}$, $Q_{HSI:DYAb}$	Glutathione S-transferase IV; Uncharacterized protein
GRMZM2G376061	3	$Q_{HSI:DYb}$	no information

Continued on next page

Gene	Chr	QTL	Description
GRMZM2G042278	5	$Q_{HSI:MFb}$, Q_{PC1b}	Uncharacterized protein
GRMZM2G048672	5	$Q_{HSI:MFb}$, Q_{PC1b}	Macrophage migration inhibitory factor
GRMZM2G048904	5	$Q_{HSI:MFb}$, Q_{PC1b}	Alpha-L-fucosidase 2
GRMZM2G059124	5	$Q_{HSI:MFb}$, Q_{PC1b}	no information
GRMZM2G064360	5	$Q_{HSI:MFb}$, Q_{PC1b}	Basic endochitinase 1
GRMZM2G066578	5	$Q_{HSI:MFb}$, Q_{PC1b}	Uncharacterized protein
GRMZM2G067306	5	$Q_{HSI:MFb}$	5S rRNA binding protein
GRMZM2G087495	5	$Q_{HSI:MFb}$, Q_{PC1b}	Uncharacterized protein
GRMZM2G089836	5	$Q_{HSI:MFb}$, Q_{PC1b}	Beta-fructofuranosidase 1; Invertase
GRMZM2G100412	5	$Q_{HSI:MFb}$	no information
GRMZM2G102862	5	$Q_{HSI:MFb}$, Q_{PC1b}	no information
GRMZM2G120539	5	$Q_{HSI:MFb}$, Q_{PC1b}	AMP binding protein
GRMZM2G128938	5	$Q_{HSI:MFb}$, Q_{PC1b}	no information
GRMZM2G133262	5	$Q_{HSI:MFb}$, Q_{PC1b}	no information
GRMZM2G133434	5	$Q_{HSI:MFb}$, Q_{PC1b}	Peroxidase 45
GRMZM2G144420	5	$Q_{HSI:MFb}$, Q_{PC1b}	no information
GRMZM2G162093	5	$Q_{HSI:MFb}$, Q_{PC1b}	Uncharacterized protein
GRMZM2G165308	5	$Q_{HSI:MFb}$, Q_{PC1b}	Uncharacterized protein
GRMZM2G168747	5	$Q_{HSI:MFb}$, Q_{PC1b}	no information
GRMZM2G173596	5	$Q_{HSI:MFb}$, Q_{PC1b}	ZIM motif family protein
GRMZM2G177863	5	$Q_{HSI:MFb}$, Q_{PC1b}	Uncharacterized protein
GRMZM2G305446	5	$Q_{HSI:MFb}$, Q_{PC1b}	Aquaporin TIP3-1
GRMZM2G340282	5	$Q_{HSI:MFb}$, Q_{PC1b}	no information
GRMZM2G375607	5	$Q_{HSI:MFb}$, Q_{PC1b}	no information
GRMZM2G413006	5	$Q_{HSI:MFb}$, Q_{PC1b}	Xyloglucan endotransglucosylase/hydrolase protein 23
GRMZM2G414159	5	$Q_{HSI:MFb}$, Q_{PC1b}	Uncharacterized protein
GRMZM2G436710	5	$Q_{HSI:MFb}$, Q_{PC1b}	Uncharacterized protein
GRMZM2G439195	5	$Q_{HSI:MFb}$	Nicotianamine synthase 3
GRMZM2G474555	5	$Q_{HSI:MFb}$, Q_{PC1b}	Putative uncharacterized protein
AC195914.2_FG003	5	Q_{PC1b}	Uncharacterized protein
GRMZM2G002240	5	Q_{PC1b}	no information
GRMZM2G419455	5	Q_{PC1b}	no information
GRMZM2G019872	9	$Q_{HSI:MFc}$, $Q_{HSI:LS}$	NADP-dependent oxidoreductase P2; Putative alcohol dehydrogenase superfamily protein
GRMZM2G051135	9	$Q_{HSI:MFc}$, $Q_{HSI:LS}$	Uncharacterized protein
GRMZM2G056093	9	$Q_{HSI:MFc}$, $Q_{HSI:LS}$	Uncharacterized protein
GRMZM2G113203	9	$Q_{HSI:MFc}$, $Q_{HSI:LS}$	Uncharacterized protein
GRMZM2G126900	9	$Q_{HSI:MFc}$, $Q_{HSI:LS}$	Uncharacterized protein
GRMZM2G133050	9	$Q_{HSI:MFc}$, $Q_{HSI:LS}$	Uncharacterized protein
GRMZM2G145446	9	$Q_{HSI:MFc}$, $Q_{HSI:LS}$	Uncharacterized protein
GRMZM2G163178	9	$Q_{HSI:MFc}$, $Q_{HSI:LS}$	Uncharacterized protein
GRMZM2G166459	9	$Q_{HSI:MFc}$, $Q_{HSI:LS}$	Putative MATE efflux family protein; Uncharacterized protein
GRMZM2G404603	9	$Q_{HSI:MFc}$, $Q_{HSI:LS}$	Putative uncharacterized protein 9C20.6a; Uncharacterized protein
GRMZM2G704251	9	$Q_{HSI:MFc}$, $Q_{HSI:LS}$	Uncharacterized protein
GRMZM5G844143	9	$Q_{HSI:MFc}$, $Q_{HSI:LS}$	Photosystem Q(B) protein
GRMZM2G043338	9	$Q_{HSI:LS}$	Uncharacterized protein
GRMZM2G043955	9	$Q_{HSI:LS}$	Uncharacterized protein
GRMZM2G071074	9	$Q_{HSI:LS}$	Uncharacterized protein
GRMZM2G094990	9	$Q_{HSI:LS}$	Beta-expansin 1a; Uncharacterized protein
GRMZM2G132238	9	$Q_{HSI:LS}$	Putative metacaspase family protein

Continued on next page

Gene	Chr	QTL	Description
GRMZM2G149273	9	$Q_{HSI:LS}$	Peroxidase K
GRMZM2G178645	9	$Q_{HSI:LS}$	Uncharacterized protein
GRMZM2G384661	9	$Q_{HSI:LS}$	Uncharacterized protein
GRMZM2G024718	9	$Q_{HSI:LS}$	Heat shock protein 1; Uncharacterized protein
GRMZM2G036351	9	$Q_{HSI:LS}$	Putative tify domain/CCT motif transcription factor family protein; ZIM motif family protein
GRMZM2G051689	9	$Q_{HSI:LS}$	CRAL/TRIO domain containing protein; Uncharacterized protein
GRMZM2G067225	9	$Q_{HSI:LS}$	Putative cytochrome P450 superfamily protein; Uncharacterized protein
GRMZM2G078469	9	$Q_{HSI:LS}$	Putative DUF231 domain containing family protein; Uncharacterized protein
GRMZM2G078472	9	$Q_{HSI:LS}$	Asparagine synthetase
GRMZM2G113060	9	$Q_{HSI:LS}$	Putative AP2/EREBP transcription factor superfamily protein; Uncharacterized protein
GRMZM2G154316	9	$Q_{HSI:LS}$	Uncharacterized protein
GRMZM2G330945	9	$Q_{HSI:LS}$	Uncharacterized protein
GRMZM2G366532	9	$Q_{HSI:LS}$	Uncharacterized protein
GRMZM2G479260	9	$Q_{HSI:LS}$	Class I heat shock protein 3; Uncharacterized protein
GRMZM2G586702	9	$Q_{HSI:LS}$	Uncharacterized protein
